# Preclinical and clinical advances in transposon-based gene therapy

**DOI:** 10.1042/BSR20160614

**Published:** 2017-12-05

**Authors:** Jaitip Tipanee, Yoke Chin Chai, Thierry VandenDriessche, Marinee K. Chuah

**Affiliations:** 1 Department of Gene Therapy and Regenerative Medicine, Free University of Brussels (VUB), Brussels, Belgium; 2 Department of Cardiovascular Sciences, Center for Molecular and Vascular Biology, University of Leuven, Leuven, Belgium

**Keywords:** induced pluripotent stem cells, piggyBac, Sleeping Beauty, stem cells, transposon, Tol2

## Abstract

Transposons derived from *Sleeping Beauty* (SB), *piggyBac* (PB), or *Tol2* typically require cotransfection of transposon DNA with a transposase either as an expression plasmid or mRNA. Consequently, this results in genomic integration of the potentially therapeutic gene into chromosomes of the desired target cells, and thus conferring stable expression. Non-viral transfection methods are typically preferred to deliver the transposon components into the target cells. However, these methods do not match the efficacy typically attained with viral vectors and are sometimes associated with cellular toxicity evoked by the DNA itself. In recent years, the overall transposition efficacy has gradually increased by codon optimization of the transposase, generation of hyperactive transposases, and/or introduction of specific mutations in the transposon terminal repeats. Their versatility enabled the stable genetic engineering in many different primary cell types, including stem/progenitor cells and differentiated cell types. This prompted numerous preclinical proof-of-concept studies in disease models that demonstrated the potential of DNA transposons for *ex vivo* and *in vivo* gene therapy. One of the merits of transposon systems relates to their ability to deliver relatively large therapeutic transgenes that cannot readily be accommodated in viral vectors such as full-length dystrophin cDNA. These emerging insights paved the way toward the first transposon-based phase I/II clinical trials to treat hematologic cancer and other diseases. Though encouraging results were obtained, controlled pivotal clinical trials are needed to corroborate the efficacy and safety of transposon-based therapies.

## Introduction

Viral vectors are most commonly used for clinical gene therapy resulting in the marketing authorization approval for several products to treat genetic diseases and cancer [1,2]. Despite their promise, the main limitation is their immunogenicity when administered directly *in vivo*, that may curtail long-term gene expression and/or prevent vector re-administration. Moreover, integrating viral vector carries a risk of insertional oncogenesis or clonal dominance [3–7]. In addition, the maximum cargo size of viral vectors is restricted, hampering gene transfer of larger therapeutic genes. Finally, viral vector manufacturing is cumbersome and costly and requires living cells for their production which complicates the quality control and downstream processing. As an alternative, non-viral transfection methods have been considered to circumvent some of these limitations.

Typically, non-viral transfection requires the introduction of plasmids harboring the transgene expression cassettes into the desired target cells, in the absence of any viral elements. To enhance somatic gene expression for therapeutic purposes, non-viral vector gene therapy can be achieved by either adding functional gene copies (referred to as ‘gene addition’) or correcting non-functional mutated genes in the target genome (referred to as ‘gene correction’). Gene delivery can be achieved *ex vivo*, whereby donor-derived cells are cultured and genetically modified *in vitro* and subsequently reintroduced to autologous or allogeneic recipients [8]. Alternatively, *in vivo* gene transfer is applied locally or systemically to genetically modify the target cells *in situ* [9]. A variety of non-viral gene delivery methods have been developed to improve the efficacy of gene transfer. However, non-viral vectors do not lead to DNA integration of the gene of interest into the host genome. Consequently, this may ultimately compromise long-term gene expression due to degradation of the non-integrated episomes and/or dilution upon cell proliferation. This justifies the use of transposable elements, that are able to insert themselves in the target genome for gene therapy applications [9–13]. Non-viral transfection of transposons not only enables stable expression due to their genomic integration capabilities but also diminishes the risk of immunogenicity. Moreover, the non-viral transfection components are entirely synthetic enabling cell-independent vector manufacturing, significantly lowering the production costs.

## DNA transposons

DNA transposons translocate via a non-replicative, ‘cut-and-paste’ mechanism. This requires recognition of the two terminal inverted repeats (TIRs) by a DNA transposase that cleaves its target and consequently releases the DNA transposon from its donor template. Upon excision, the DNA transposons subsequently integrate into the acceptor DNA that is cleaved by the same transposase. Typically, this results in target-site duplications (TSDs) at the insertion sites [9]. There are evolutionary remnants of transposon DNA in the human genome but they have become silent during evolution and in principle are unable to undergo transposition [14]. This minimizes concerns associated with endogenous transposon remobilization and genome instability. In their natural configuration, DNA transposons are flanked by two TIRs and contain a gene encoding a transposase that catalyzes transposition. There are different categories of DNA transposons that differ with respect to their actual DNA sequence, target site recognition, TSDs, and TIRs. Typically, transposases belonging to one particular category cannot catalyze transposition of another type of transposon.

For gene transfer applications with DNA transposons, it was necessary to develop a binary system based on two distinct plasmids whereby the transposase was physically separated from the transposon DNA containing the gene of interest flanked by the TIRs. Co-delivery of the transposon and transposase plasmids into the target cells enables transposition via a conventional cut-and-paste mechanism [9]. Ideally, the transposase should be expressed for a short time only and sustained expression should be avoided as this may lead to continuous transposon mobilization and integration. However, it is important to minimize the number of vector copies per cell as this increases the risk of insertional oncogenesis. Typically, the expression plasmid encoding the transposase should gradually disappear from the transfected cells due to DNA degradation and/or dilution upon cell division. However, even non-integrated expression plasmids could give rise to low-level continuous expression of the transposase. Therefore, it cannot be excluded that these expression plasmids encoding the transposase may potentially integrate providing a continuous source of transposase expression. Moreover, sustained transposase expression could potentially result in continuous transposon remobilization and integration. As a safer alternative, it is possible to also deliver the transposase as an mRNA [15,16] that results in its transient expression sufficient to enable transposition while minimizing the risk of insertional oncogenesis.

Transposase acts as a catalytic enzyme to enable transposition process through cut-and-paste mechanism. However, excess transposase concentration may lead to reduction in transposition activity (referred as ‘overproduction inhibition’), potentially via the formation of transposase multimers which are functionally inactive. This phenomenon has been observed in some transposon systems [17,18]. Hence, it is crucial to optimize the dose of transposase/transposon ratio upon gene transfer in order to achieve the highest integration efficiency. In addition, it is possible to alleviate overproduction inhibition effect and enhance transposition activity by specific transposase–DNA binding domain protein fusion. Perhaps this increases the overall affinity and specificity of modified transposase toward target DNA [19].

The plasmids containing the transposon and transposase also harbor bacterial sequences such as the origin of replication and the bacterial selection marker. This adversely affects the size of the transfected DNA and serves no purpose in the eukaryotic target cells. It would be also preferred to eliminate these sequences in order to prevent their inadvertent introduction into the target cells, and to increase the overall transfection efficiency and reduce potential cellular toxicity by lowering the amount of DNA introduced into the cells [20–22].

Different types of transposons have been considered for human gene therapy applications [9,10]. In this review, we have selected three types of transposons that are widely used in the field: (i) *Sleeping Beauty* (SB), (ii) *piggyBac* (PB), and (iii) *Tol2* transposons, and their biological characteristics including the species of origin, classification, molecular structure, cargo capacity, DNA integration profile as described below and summarized in Table 1.

**Table 1:** Comparison of SB, PB and *Tol2* features

Characteristics	SB	PB	*Tol2*
**(i) Species of origin**	Salmonid fish [23,24]	Cabbage looper moth [35,36]	Medaka fish [48]
**(ii) Classification**	*Tc1/mariner* superfamily [23,24]	*PB* superfamily [35,36]	*hAT* superfamily [48]
**(iii) Molecular structure**	~1.6 kb in length	~2.5 kb in length	~4.7 kb in length
	Comprises two inverted repeats/direct repeats	Comprises outer symmetrical and inner asymmetrical TIRs	Comprises two TIRs
	Transposase is 360 amino acids in length [9]	Transposase is 594 amino acids in length [37]	The most active transposase is 649 amino acid in length [51]
**(iv) Target site preference**	AT [155]	TTAA [156]	8-bp random nucleotides (potentially has weak consensus sequence (C/G)TTATAA(G/C)) [51,157]
**(v) Transposon footprint**	Yes [170]	No [171]	Yes [172,173]
**(vi) Cargo capacity**	Up to 12 kb [28]	Up to 200 kb [174]	Up to 11 kb
**(vii) Local hopping**	Yes	Yes	Yes
	(~30–60% within donor chromosome) [175–177]	(~9–30% within donor chromosome) [178,179]	(~20% within donor chromosome) [61,157]
**(viii) Overproduction inhibition**	Yes [17,180,181]	Yes [17,18]	Yes [17]
**(ix) DNA integration profile**	~25–45% in RefSeq genes	~50–55% in RefSeq genes	~40% in RefSeq genes
	<2% in TSSs	~2–20% in TSSs	~2–8% in TSSs
	~2% in CpG islands	~4–18% in CpG islands	~4–13% in CpG islands
	<1% in DNase I hypersensitivity regions [83,128,182]	~1–5% in DNase I hypersensitivity regions [128,156]	~1–5% in DNase I hypersensitivity regions [128,157]

Abbreviation: hAT, hobo/Ac/Tam3; TSS, transcriptional start site.

### SB

**Figure 1:**
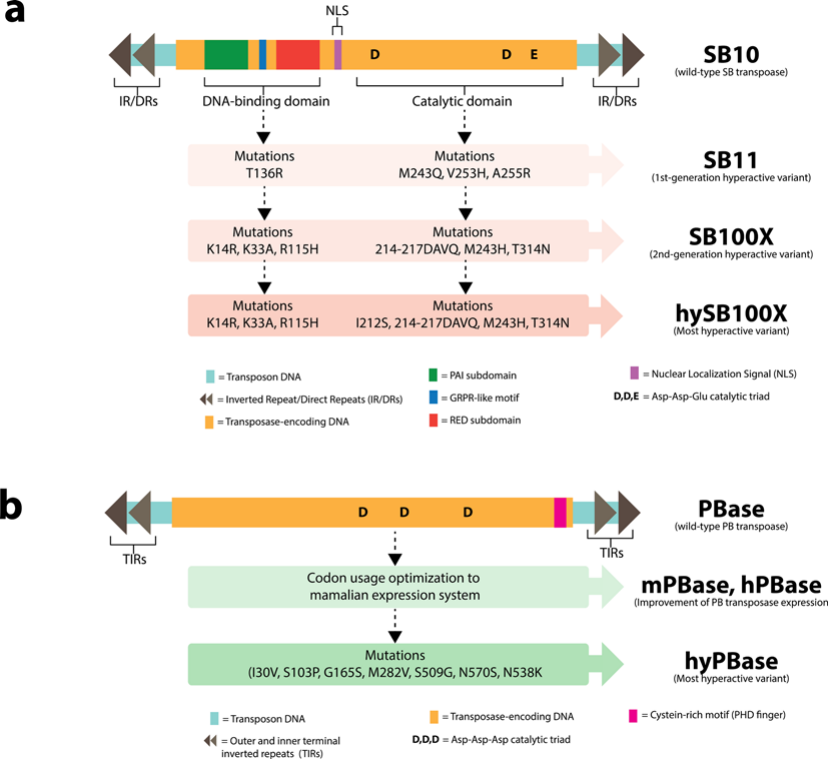
Molecular architecture and transposase evolution of *SB* and *PB* systems for gene delivery (**a**) SB *transposon* is ~1.6 kb in total length and consists of two inverted repeat/direct repeats (IR/DRs) flanking DNA encoding transposase [9]. After resurrection from fish genomes, the native functional SBase (SB10) has 360 amino acids, which can be divided into DNA-binding domain (DBD) and catalytic domain. The DBDs contain two helix-turn-helix subdomains (PAI and RED subdomains) separated by GRPR-like motif [165]. The conserved Asp-Asp-Glu (DDE) trinucleotide is present in catalytic domain for DNA cleavage upon transposition [166]. SBase has undergone molecular evolution through amino acid substitutions to improve transposition efficiency for gene transfer, giving rise to more active SBase mutants such as SB11 and SB100X. The most hyperactive variant of SBase by far is ‘hySB100X’, which increases 30% of transposition rate compared with SB100X [32]. (**b**) PB *transposon* is ~2.5 kb in total size and carries two outer and inner TIRs at the end of transposon. DNA flanked by TIRs encodes 594-amino acid PB transposase (PBase) [37]. The detailed structure of PBase relatively remains elusive; however, it possesses Asp-Asp-Asp (DDD) catalytic triad for transposition [167]. Cysteine-rich motif is located at C-terminus and suggested to form plant homeodomain (PHD) finger [168]. Bipartite nuclear localization signal (NLS) is recently identified at this region [169]. Two major approaches have been employed to enhance transposition efficacy upon gene delivery: (i) *codon usage optimization* corresponding to mammalian hosts to promote transposase expression within the cells (i.e. mouse PBase (mPBase) and human PBase (hPBase)), and (ii) hyperactive transposase variant screening by error-prone PCR (i.e. hyperactive PBase (hyPBase)). *In vivo* comparative study indicates superior transposition activity of hyPBase compared with mPBase by increasing transgene expression up to 100-fold [43].

The SB transposon is a member of the *Tc1/mariner* superfamily and shares homology with the *Caenorhabditis elegans Tc1* transposon. The SB transposon was initially identified in fish DNA [23]. Since it had acquired inactivating mutations in the transposase gene, it was unable to undergo transposition. To convert the SB transposon into a tool for stable gene transfer, it was therefore necessary to reconstruct a functional transposase (SB10) by ‘*reverse evolution’* based on consensus DNA sequences of different transposons derived from different fish species [24]. These pioneering efforts showed that the reverse-engineered SB transposase (SBase) could catalyze transposition of its cognate transposon DNA [24]. Nevertheless, the overall transposition efficiency remained low and insufficient for most gene therapy clinical applications. To overcome this limitation, hyperactive SBase mutants have been generated by introducing specific mutations into the original SB10 transposase, such as SB11 [25], HSB5 [26–28], and SB100X [29] (Figure 1). The SB100X transposase is a highly efficient hyperactive SBase resulting in a 100-fold increase in transposon mobilization efficiency than the original SB10 based on a marker rescue assay. This SB100X distinguishes itself from the other SBases by the fact that it was generated by evolution and selection *in vitro*. The increased transposition efficiency of the SB100X system makes it particularly attractive for gene therapy applications in clinically relevant primary cells, such as CD34^+^ hematopoietic stem cell (HSC), muscle stem/progenitor cells, mesenchymal and mesoangioblasts [29–31]. In addition, SB100X system efficiently enables stable gene transfer in induced pluripotent stem (iPS) cells and its differentiated derivatives [31]. Through a structure-based design and molecular engineering approach, Voigt et al. [32] recently reported a novel hyperactive variant SBase called ‘hyperactive SB100X’ (hySB100X), which exhibited a 30% significantly higher transposition rate compared with SB100X. This higher transposition rate was achieved by introducing a mutation (I212S) to short hydrophilic residues in the catalytic domain of the SB100X transposase molecule which conferred direct DNA contact and thus increased the transposon DNA binding affinity.

The SB systems have been employed to readily deliver transgenes of up to 8 kb in size, though transposition efficiency decreased with an increase in transgene size beyond 8 kb [25]. However, the transposon payload could be increased further by either flanking the expression cassette with two complete pairs of inverted repeat/direct repeats (IR/DRs) in inverted orientation (i.e. ‘*sandwich configuration*’) [33] or by using viral vectors such as herpes simplex virus (HSV) amplicon vector [34] or a helper-dependent adenoviral vector [28] for delivering the transposon components.

### PB

The PB transposon was discovered in genome of cabbage looper moth *Trichoplusia ni* [35–39]. Similar to SB, PB transposon relies on non-replication mechanism during transposition and generates TSDs at the end of the process. Several improvements in PB system have been employed to augment transposition efficiency. For instance, DNA sequence of PB transposase (PBase) has been optimized based on codon usage corresponding to host systems. This gives rise to mouse (mPBase) and human (hPBase) codon-optimized PBase that efficiently express in mammalian cells and have an improved transposition rate [40,41]. Another approach is to introduce the novel amino acid mutations into wild-type PBase and screen for hyperactive variants with increased transposition activity (Figure 1). The most hyperactive PBase (hyPBase) by far was engineered to harbor seven amino acid substitutions and improve transposition efficiency up to 20-fold relative to mPBase [42,43]. Apart from transposase engineering, truncation of TIR length could be implemented to minimize the total size of PB transposon, thereby promoting transposition efficiency upon gene delivery [44]. This ultimately yields the most compact 5′ and 3′ TIRs (referred to as ‘IR_micro_’), which contributed to 1.5-fold improvement in transgene expression *in vivo* compared with wild-type TIRs [43]. In addition, TIR variant carrying T53C and C136T nucleotide substitution (referred to as ‘IR_mut’_) significantly increased transposition rate by 59% when used in conjunction with hPBase (referred to as ‘enhanced *PB* system’) [40]. However, the effect of IR_mut_ on transposition does not differ from native TIRs based on mouse study [43].

Another superior feature of PB over SB and viral vector systems is its capability to deliver up to 100 kb of DNA cargo [45–47], and modified PB transposon can mediate gene delivery of 200-kb bacterial artificial chromosome (BAC) for generation of transgenic models [47]. This signifies the potential of PB platform to carry transgenes with relatively large size such as full-length dystrophin for efficient gene therapy, which is usually challenging by using viral vectors [30].

### Tol2

The *Tol2* transposon is derived from a medaka fish (*Orizyas latipes*) genome and categorized into *hAT (hobo/Ac/Tam3)* superfamily [48]. As a tool for gene therapy, *Tol2* system can be employed to achieve long-term transgene expression [49,50]. In addition, codon-usage optimization of *Tol2* transposase for mammalian gene expression was generated to enhance transposition efficiency up to three-fold compared with native transposase [51,52]. Alternatively, the total size of *Tol2* vector was curtailed by shortening 5′ and 3′ ends of transposon backbone (i.e. ‘minimal *Tol2’*), and this gave rise to approximately three-fold improvement in transposition efficacy relative to original *Tol2* transposon [53]. Similar to SB, *Tol2* transposon can readily accommodate up to 11 kb transgene for efficient transposition [49,50,54,55]. In addition, the successful use of *Tol2* system has been extensively demonstrated in a number of transgenesis studies, prominently in zebrafish [49,52,56–62].

Collectively, SB, PB, and *Tol2* systems allow efficient non-viral gene transfer for sustained gene expression with large cargo capacity. However, it is essential to assess the relative potential of these transposon systems in order to achieve the most efficient gene transfer method in different contexts of study models. A variety of confounding factors (e.g. transfection efficiency, proportion of transposon/transposase etc.) can substantially influence transposition efficacy upon gene delivery, so it is crucial to adjust all relevant parameters to the same setting during cross-comparison. For example, side-by-side comparative study of human CD34^+^ HSCs showed that SB100X mediated four-fold greater transposition activity than mPBase. Moreover, *Tol2*-based transposon system for gene transfer is relatively inefficient compared with mPBase and SB100X [17]. Thus, the proper selection of transposon system for further clinical use is considered one of the major keys to maximize the efficiency of gene transfer.

## Preclinical advances in transposon-based gene therapy

A number of transfection methods have been utilized to direct therapeutic gene expression in clinically relevant cells for disease treatment. These approaches differ in terms of mechanism of transfection and cytotoxicity, ultimately yielding diverse efficacy in gene transfer. In addition, intrinsic properties of each cell type and total size of transgene expression cassette could influence transfection rate. Chemical-based transfection methods such as calcium phosphate, polyethyleneimine (PEI), and cationic liposomes are typically used for transfection in common cell lines [63–68], and high efficiency of gene transfer could be obtained. Generally, these methods allow the formation of DNA–chemical complex via electrostatic interaction and enhance DNA uptake in host cells by endocytosis or membrane fusion. Alternatively, electroporation is known for high-efficiency transfection technique by creating transient pores in cell membranes for DNA transfer. This method is employed for transfection in most primary cells and suspension cells which are comparably intractable with chemical-based transfection [29–31,50,69]. However, electroporation often compromises cell viability due to cellular stress caused by DNA toxicity and extreme conditions during transfection.

*In vivo* gene therapy could also be achieved by the transposon system. Hydrodynamic (HD) injection is considered one of the most common methods for *in vivo* transposon delivery through high-speed injection of large volume of DNA solution. This approach can be assisted by catheter and has been successfully demonstrated in a broad range of animal models including mouse [43,70], rat [71,72], rabbit [73], pig [74], dog [75,76], and monkey [44]. HD injection primarily targets gene transfer in liver; however, *in vivo* kidney and muscle transfection can also be attained using this method. In addition, PEI-based transfection by intravenous injection has been validated for transposon delivery in murine model, but it yielded relatively low efficiency of gene transfer compared with HD injection. As an alternative, nanoparticles harboring transposons effectively mediate gene transfer in liver sinusoidal endothelial cells and primary T cells in mice [77,78]. The comparable efficiency of gene delivery mediated by *ex vivo* and nanoparticle-based *in vivo* transfection was observed in primary T cells, obviating the need of T-cell isolation for *in vitro* genetic engineering [78]. Moreover, transposons can also be encapsidated in viral vectors such as adenoviral [28,79–81], adeno-associated viral (AAV) [80], herpes simplex viral [82], and integration-deficient lentiviral vectors [83–85] to overcome cellular barriers and enhance gene transfer rate in mouse and dog models [79,80,82]. Taken together, this demonstrates the advancement of systemic *in vivo* delivery of transposon for therapeutic purposes. Previous studies reported intramuscular electroporation of transposons in murine muscle in order to mediate localized transgene expression; however, it did not contribute to long-term gene expression [68]. Hence, further development is needed to optimize the method for local delivery of transposon in muscle tissue.

A number of clinically relevant cells and organs have been successfully demonstrated targetted by SB- and PB-based gene therapy in several disorders. This has been summarized in Table 2 (for SB transposons) and Table 3 (for PB transposons). The study of *Tol2* transposon for gene therapy is relatively limited compared with SB and PB transposons and by far has been explored only in primary T cells for adoptive therapy [50]. Several specific target diseases are highlighted and discussed in more detail below.

**Table 2:** SB transposon application in gene therapy research

Target cells/organs	Diseases	Species of cell origin	Therapeutic genes	Gene transfer approach	Improvements			Remarks	References
					Transposon	Transposase	Others		
*Clinically relevant cells*									
(i) Epidermal cell	Junctional epidermolysis bullosa	Human	*Laminin β3*	Chemical transfection				*Ex vivo* therapy in mouse	[183]
(ii) HSC	SCD	Human	*β-globin*	Electroporation		SB100X			[94,95]
(iii) Hepatoma cell lines	Hepatocellular carcinoma	Human	*HSV thymidine kinase*	Chemical transfection					[184]
(iv) Keratinocyte	Epidermolysis bullosa	Human	*Type VII collagen*	Chemical transfection		SB100X		*Ex vivo* therapy in mouse	[185]
(v) Medulloblastoma cell line	Huntington’s disease	Human	*Huntingtin-specific siRNA*	Chemical transfection					[186]
(vi) MSC	Mucopolysaccharidosis	Mouse	*α-L-iduronidase*	Electroporation		SB100X		*Ex vivo* therapy in mouse	[101]
(vii) Myoblast	Limb-girdle muscular dystrophy	Mouse	*Dysferlin*	Electroporation		SB100X			[110]
(viii) Lymphoblastoid cell	Type C Fanconi anemia	Human	*Fanconi anemia*	Electroporation		SB100X			[187]
(ix) Retinal pigment epithelium	Alzheimer’s disease	Human	*Nerve growth factor*	Chemical transfection		SB100X		*Ex vivo* therapy in rat/human	[188,189]
	Age-related macular degeneration	Rat	*Pigment epithelium derived factor*	Electroporation		SB100X		*Ex vivo* therapy in rat	[20]
(x) Satellite cell	Duchenne muscular dystrophy	Mouse	*Microdystrophin*	Electroporation		SB100X		*Ex vivo* therapy in mouse	[109]
(xi) T lymphocyte	B-lineage malignancies	Human	*Chimeric antigen receptor*	Electroporation		SB11		*Ex vivo* therapy in mouse/human	[127,129,133,135]
						SB100X			
*Organs*									
(i) Brain	Glioblastoma	Mouse	*Interferon-γ*	Local infusion + PEI		SB13			[190]
(ii) Hepatic endothelial cell	Hemophilia A	Mouse	*Factor VIII*	Local IV + PEI		HSB17		One-component system	[77,120]
				Nanocapsule IV					
(iii) Liver	Colorectal cancer	Mouse	*Angiostatin-endostatin fusion*	HD		SB11			[191]
	Type I Crigler–Najjar syndrome	Rat	*Uridinediphosphoglucuronate glucuronosyltransferase-1A1*	FPL		HSB3			[192]
	Type I diabetes	Mouse	*Insulin*	HD					[193]
	Familial hypercholesterolemia	Mouse	*Low-density lipoprotein and very-low-density lipoprotein receptors*	HD		SB100X			[194]
	Hemophilia A	Mouse	*Factor VIII*	HD		HSB16			[116,117]
			*Indoleamine 2,3-dioxygenase*						
	Hemophilia B	Mouse	*Factor IX*	IV (+ hybrid adenoviral vector)		HSB5			[29,79,118,182,195]
		Dog				SB100X			
	Mucopolysaccharidosis	Mouse	*β-glucuronidase*	HD		SB11	One-component SB system		[76,102–104]
		Dog	*α-L-iduronidase*			SB100X			
	SCD	Mouse	*Heme oxygenase-1*	HD		SB100X			[96–98]
			*Hemopexin*						
			*Ferritin heavy chain ferroxidase*						
	Thrombotic thrombocytopenic purpura	Mouse	*A disintegrin and metalloproteinase with thrombospondin type 1 motif, member 13*	HD		SB100X			[196]
	Tyrosinemia type I	Mouse	*Fumarylacetoacetate hydrolase*	HD		SB11	mRNA as a source of SBase		[197–199]
(iv) Lung	Pulmonary fibrosis	Rat	*Indoleamine-2,3-dioxygenase*	IV + PEI		SB11			[200,201]
		Mouse	*miR-29*			HSB17			
(v) Pulmonary endothelium	Pulmonary hypertension	Rat	*Indoleamine-2,3-dioxygenase*	IV + PEI		HSB16			[72,202]
		Mouse	*Nitric oxide synthase*			HSB17			

*If not specifically indicated, two-component SB transposon system was used in conjunction with wild-type SBase (SB10). Abbreviations: FPL, fusogenic galactose-terminated F-glycoprotein of the Sendai virus; HSC, hematopoietic stem cell; IV, intravenous injection; MSC, mesenchymal stem cell; SCD, sickle cell disease.

**Table 3:** PB transposon application in gene therapy research

Target cells/organs	Diseases	Species of cell origin	Therapeutic genes	Gene transfer approach	Improvements			Remarks	References
					Transposon	Transposase	Others		
*Clinically relevant cells*									
(i) Induced pluripotent stem cell	α1-antitrypsin deficiency	Human	*α-1 antitrypsin*	Electroporation		hyPBase	Use with ZFN		[203]
	β-thalassemia	Human	*β-globin*	Electroporation		hyPBase	Use with CRISPR/Cas9		[147]
	SCD	Human	*β-globin*	Electroporation		hyPBase	Use with TALEN		[146]
(ii) Mesoangioblast	Duchenne muscular dystrophy	Dog	*Microdystrophin*	Electroporation		hyPBase			[30]
(iii) T lymphocyte	Hematological malignancies, HER2-specific cancer	Human	*Chimeric antigen receptor*	Electroporation		hyPBase			[130,131,204–206]
*Organs*									
(i) Kidney	Unilateral ureteral obstruction	Mouse	*Insulin-like growth factor-1 receptor*	HD					[207,208]
			*Glutathione transferase isozyme A4*						
(ii) Liver	Hemophilia A	Mouse	*Factor VIII*	HD		hyPBase			[70,122]
	Hemophilia B	Mouse	*Factor IX*	HD	IR_mut_	mPBase			[43,121]
				Ultrasound + microbubble	IR_micro_	hyPBase			
	von Willebrand disease	Mouse	*von Willebrand factor*	HD		hyPBase			[209]
(iii) Solid tumor	Cervical and ovarian cancer	Mouse	*HSV thymidine kinase*	Local infusion + PEI					[210,211]

*If not specifically indicated, two-component PB transposon system was used in conjunction with wild-type PBase. Abbreviations: CRISPR/Cas9, clustered regularly interspaced short palindromic repeats/CRISPR-associated protein-9 nuclease; HSV, herpes simplex virus; MAB, mesoangioblast; SCD, sickle cell disease; TALEN, transcription activator like effector nuclease; ZFN, zinc finger nuclease.

### Sickle cell disease

Sickle cell disease (SCD) is caused by single amino acid substitution in hemoglobin gene called β-globin, which gives rise to sickling phenotype of erythrocytes during deoxygenation. These sickle cells subsequently form aggregates and thus impede circulatory flow within body and ultimately lead to organ damage as well as inflammation [86]. In addition, the sickling morphology of red blood cells contributes to hemolysis and leads to leakage of heme and hemoglobin into the circulation. Moreover, heme is known to act as a proinflammatory agent, and its excess amount potentially promotes renal injury. To neutralize its toxic effects, typically free heme is trapped by hemopexin (Hpx) and engulfed into hepatocytes and macrophage [87]. Alternatively, heme can be metabolized by heme oxygenase-1 enzyme into ferrous (Fe^2+^), carbon monoxide (CO), and biliverdin [88], and ferritin heavy chain ferroxidase subsequently catalyzes Fe^2+^ conversion into Fe^3+^ – a non-toxic form of Fe [89]. The levels of serum proteins which are responsible for heme scavenging in patients with SCD were substantially different from those in healthy adults [90,91]. Therefore, these heme-binding proteins may serve as a therapeutic target for SCD treatment.

HSCs are considered potential candidates for gene therapy in SCD as they are capable of self-renewal and multilineage differentiation into various blood cell types. The first genetic modification in CD34^+^ HSCs by transposons was demonstrated using the original SB (SB10) system. Subsequently, improved versions of SBase were employed to boost transgene expression in HSCs [92,93]. These studies reported that the transposition efficiency in HSCs could be enhanced to 4% by HSB16, and even up to 30% by the hyperactive SBase SB100X [92,93]. It is particularly encouraging that the genetically engineered HSCs following SB-mediated gene delivery could stably express transgenes and undergo cellular differentiation into the typical hematopoietic lineages. For several decades, stable and efficient non-viral gene modification of HSC capable of hematopoietic reconstitution remained an elusive goal. This prompted the use of integrating viral vectors as an alternative for genetic engineering of CD34^+^ HSC. However, our studies using SB100X demonstrated, for the first time, that the SB-modified CD34^+^ HSCs enabled hematopoietic reconstitution in mice following cell engraftment with gene marking in both lymphoid and myeloid lineages [29,93]. This opens new perspectives for the use of non-viral transposon-based transfection methods for HSC applications, including SCD. Nevertheless, there is still a need to augment transfection efficiencies in CD34^+^ HSC while minimizing potential toxicities.

Alternatively, the PB system has also been employed to direct gene transfer in CD34^+^ HSCs, but comparative analysis indicated the superior activity of SB100X compared with mPBase [17]. It would be interesting to conduct head-to-head comparative studies between the most hyperactive variants of SBase (hySB100X) and PBase (hyPBase). As a tool for gene therapy in SCD, the SB100X system was employed to introduce the *β-globin* gene into patient-derived CD34^+^ HSCs [94,95]. The transposon-modified HSCs continuously expressed functional globin chain protein and exhibited an amelioration of the sickling phenotype and disease progression [94]. This underscores the clinical potential of transposon-modified CD34^+^ HSCs for *ex vivo* gene therapy of SCD.

Besides *ex vivo* genetic modification in CD34^+^ HSC, a transposon-based *in vivo* gene transfer strategy has also been developed to overexpress therapeutic genes to overcome some of the SCD-associated complications. In particular, SB10 was shown to mediate stable hepatic expression of heme oxygenase-1. Consequently, this alleviated vascular stasis in a murine model of SCD [96]. Later on, the efficiency of SB100X system was explored to direct ectopic expression of Hpx and ferritin heavy chain ferroxidase at therapeutic levels, which ultimately contributed to cytoprotection against vascular inflammation induced by heme in these mouse models [97,98].

### Mucopolysaccharidosis

Mucopolysaccharidosis (MPS) is a metabolic disorder characterized by the deficiency of lysosomal enzyme activity which plays a role in glycosaminoglycan (GAG) degradation. The impairment of GAG catabolism increases the abundance of GAG within lysosomes and consequently contributes to progressive tissue damage. MPS can be categorized into several subtypes based on types of affected metabolic enzymes. For instance, MPS type I is caused by the absence of α-L-iduronisase whereas deficiency in β-glucuronidase activity results in MPS type VII disease progression [99].

Mesenchymal stem cells (MSCs) have been explored as clinically relevant target cells for *ex vivo* gene therapy of MPS by virtue of their ability to evade the immune system due, in part, to the lack of expression of MHC II and co-stimulatory molecules. Moreover, similar to HSCs, MSCs exhibit self-renewal and multipotent differentiation capacities [100]. Previously, our group has successfully optimized the method of gene transfer in MSCs using the SB100X-based transposon system to achieve up to a ten-fold increase in transgene integration efficiency relative to SB11 system [31]. Subsequently, MSCs overexpressing α-L-iduronidase were generated for replacement therapy against MPS type I using the same SB100X platform. These SB100X-modified MSCs contributed to sustained expression and activity of α-L-iduronidase *in vitro*, over a 1-year time-course [101]. Direct *in vivo* administration of transposons has also been explored for MPS gene therapy in several preclinical animal models [76,102–104]. Usually this method is used in conjunction with immunomodulatory molecules, such as gadolinium chloride (GdCl_3_), to suppress immune responses in the hope to prolong expression of therapeutic enzymes. For instance, SB11 platform could elevate the hepatic expression of α-L-iduronidase and β-glucuronidase in MPS type I and VII mice, respectively [102,104], and the efficiency of transposition could be augmented up to 15-fold by SB100X [103]. Recently, the SB100X system has been validated for MPS therapy in a dog model in combination with a GdCl_3_-based immunomodulatory regimen, suggesting its therapeutic benefits for further clinical translation.

### Muscular dystrophies

Muscular dystrophies are characterized by progressive muscle degeneration, usually resulting in skeletal, smooth, and cardiac muscle dysfunction. Diverse types of muscular dystrophies have been classified according to the underlying genetic defect and disease pathology. Duchenne muscular dystrophy (DMD) is one of the most severe types of muscular dystrophies and is caused by mutations in the *dystrophin* gene. Dystrophin acts as a linker between the cytoskeleton and the extracellular matrix to support muscle integrity. Thus, myofibers are prone to damage and display a dystrophic phenotype due to the loss of dystrophin function [105]. In addition, limb-girdle muscular dystrophy type 2B and Miyoshi myopathy are characterized by dysfunction of dysferlin, the protein which is responsible for muscle repair through Ca^2+^-mediated exocytosis [106,107].

Muscle regeneration requires a sequential differentiation to repair the existing muscle fibers upon injury. Satellite cells are known as muscle progenitor cells which are capable of differentiation into myoblasts through MyoD induction, followed by subsequent differentiation of myoblasts into myotubes which can readily fuse with mature myofibers [108]. The SB100X transposon system has been employed for gene transfer in satellite cells and myoblasts to establish an *ex vivo* gene therapy for muscular dystrophy. We previously showed that ten-fold improvement of transposition efficiency could be achieved in myoblasts using SB100X instead of SB11X [31]. In addition, the SB100X system allowed us to coax myogenic differentiation of MSCs by expressing myogenic differentiation factors [31]. Moreover, the SB100X platform could efficiently contribute to stable transfer of dysferlin and microdystrophin (the truncated version of dystrophin) cDNAs in satellite cells and myoblasts and SB100X-directed engineered cells were able to engraft *in vivo* in mouse models [109,110]. Particularly, these cells can contribute to muscle regeneration and improve muscle contractile strength [109].

In contrast with myoblasts, mesoangioblasts (MABs) have the ability to migrate across the blood vessel wall and can differentiate into myogenic lineages. Transplantation of MABs into *in vivo* dystrophic models supported muscle regeneration and alleviated the severity of the disease [111–113]. This suggests the therapeutic potential of MABs for cell-based therapy in muscle disorders. Sustained expression of therapeutic genes could be achieved using PB transposons in MABs [68]. The PB system could be used to accommodate relatively large therapeutic genes, such as a non-truncated, full-length dystrophin (~11.1 kb) cDNA, to preserve all its essential functions. Recently, we have validated the PB platform in conjunction with the hyPBase to efficiently transpose the full-length codon-usage optimized human dystrophin cDNA and enable stable expression in dystrophic MABs derived from a canine dystrophy model. [30]. This study provided proof-of-concept for further development of MAB-based treatment using hyPB technology for muscular disorders.

### Hemophilia

Hemophilia A and B are X-linked recessive bleeding disorders characterized by the presence of inactivating mutations in genes encoding for blood clotting factors VIII (FVIII) and IX (FIX), respectively. Consequently, patients suffering from hemophilia A or B are highly vulnerable to spontaneous bleeding and have uncontrollable bleeding episodes upon injury. As hemophilia is a monogenic disease, gene therapy has been considered as a curative treatment for hemophilia by restoring sustained expression of the clotting factors, hence obviating the requirement of protein substitution therapy through repeated administration of factor proteins. In fact, a modest increase in plasma coagulation factor level mediated by gene therapy would be sufficient to ameliorate a severe phenotype and thus improve the clinical outcomes in hemophilia patients [114,115].

For gene therapy of hemophilia, both SB and PB systems have been used for *in vivo* expression of clotting factors in models with hemophilia. Since the liver is a known major site of clotting factor production, this organ has traditionally represented one of the main target organs for hemophilia gene therapy [43,79,116–119]. Specifically, hepatic endothelial cells such as the liver sinusoidal endothelial cells have been targetted by transposons using nanoparticles to direct FVIII expression as these cells are believed to be the endogenous source of FVIII secretion [77,120]. Alternatively, hepatocytes can be targetted with the FVIII or FIX transposon by HD transfection. Co-delivery of transposons expressing coagulation factors together with indoleamine 2,3-dioxygenase allows suppression of the immune response against the transgene products and prolong transgene expression *in vivo* [117]. Several hyperactive versions of SBase, have also been used to achieve long-term expression of FVIII and FIX, which significantly improved the bleeding diathesis in hemophilia models [29,77,79,116–120]. We demonstrated that the SB100X transposase was the most robust compared with the earlier SB versions with respect to achieving the highest sustained FIX expression levels. Alternatively, sustained hepatic expression of coagulation factors for hemophilia therapy could be attained by using the hyPB platform [43,70,121,122], which constituted a four-fold enhancement of transgene expression level compared with mPB [43]. In addition, our group showed that transposition efficiency under hyPBase activity could be substantially elevated by the use of IR_micro_ in PB vectors [43]. Similar to SB, PB technology contributed to phenotypic correction in hemophilia mice by reducing blood loss during bleeding [43,70,121,122]. In particular, *in vivo* transposon-based gene transfer for persistent expression of clotting factors (>960 days) was successfully demonstrated and had significantly shortened blood clotting time in canine model with hemophilia [79]. As an alternative, transposons encoding clotting factors have also been used for *ex vivo* gene delivery in different cell types, such as MSC [31]. One of the challenges still relates to translating this concept toward clinical applications particularly in the face of recent successes in clinical trials with AAV vectors.

### Malignancies

T lymphocytes serve as one of the major effector cells to engage and kill tumour cells through cytokine and granzyme release following T-cell activation mediated by antigen presenting cells (APCs). During activation, T lymphocytes usually recognize tumour antigen-derived peptide on MHC surface in conjunction with co-stimulatory molecules. This sometimes hampers reactive T-cell activity to efficiently target tumor cells owing to its limited capacity to broadly recognize various forms of tumor antigens [123]. Therefore, a chimeric antigen receptor (CAR)-based strategy has been developed to overcome the challenge of MHC-restricted T-cell recognition in cancer. CAR T cells are capable of direct recognition of surface foreign antigens present on tumor cells without requirement of MHC while still inducing cytolytic activity against tumor cells. The concept of CAR in cancer treatment has been largely validated and has provided significant achievement, particularly in B-cell malignancies, representing a true game-changer for leukemic cancer therapy [124].

The SB10-based transposon system was initially explored to express CD19-CARs for treating lymphoid malignancies, particularly CD19-positive B-cell leukemia [125]. The transposition efficiency for CAR T-cell production was further augmented by four-fold compared with SB11 when the SB100X technology was used [126]. Alternatively, redirecting T cells to express CD19 targetting CAR could be accomplished by using the PB and *Tol2* systems [50,78]. CAR T cells generated by transposons could efficiently lyse target cells expressing CD19 antigen and secrete cytokines for immune activation, and engraftment of redirected T cells resulted in efficient CD19-specific tumor clearance *in vivo* in preclinical tumor xenograft models [50,78,125,127]. Though wild-type PB appeared to mediate superior transposition efficacy in primary T lymphocytes compared with SB11 and *Tol2* systems [128], it is important to extend such comparative studies using the latest generation hyperactive transposases. In addition to CD19-expressing B-cell malignancy, transposon systems have been explored to redirect T cells against acute lymphocytic leukemia (ALL) [129], juvenile myelomonocytic leukemia [130], breast cancer [131], cholangiocarcinoma [132], and sarcomas [133–135].

## Generation and genetic modification of iPS using transposons

iPS cells have become a promising alternative stem cell source for treatments of many genetic diseases, including SCD. iPS cells can be generated by cellular reprogramming through introduction of *Oct4, Sox2, Klf4*, and *c-Myc* genes in somatic cells. Such iPS cells share pluripotency attributes that are indistinguishable from those of embryonic stem (ES) cells [136]. In this respect, the transposon systems appear as attractive tools for somatic cell reprogramming since they overcome some of the limitations of viral-based reprogramming technologies. In particular, the use of SB and PB platforms for iPS generation has been demonstrated in a broad range of model species starting from fibroblasts [137–143]. Indeed, comparable efficiencies of iPS generation were achieved by employing either hyPBase or SB100X system [137]. In addition, excision of gene expression cassettes from iPS genome through re-expression of transposase represents one of the unique features of transposon system for effective cellular reprogramming. It is noteworthy that the same strategy of reprogramming gene excision could also be achieved by the Cre/*loxP* recombination or clustered regularly interspaced short palindromic repeats/CRISPR-associated protein-9 (CRISPR/Cas9) technology [143,144]. This eventually gives rise to ‘transgene-free’ iPS cells, which is beneficial to minimize the risk of reactivation of reprogramming factors with oncogenic potential [144]. Nevertheless, a major advantage of PB over SB and Cre/*loxP* technology for iPS cell generation is that PB-mediated transgene removal does not leave any genetic trace in host genome, thereby providing the feasibility of seamless modification on the production of ‘genetically unmodified iPS cells’ [145]. In addition to gene addition approaches, gene correction could be achieved by a combination of PB transposons and designer endonucleases including zinc finger nuclease (ZFN), transcription activator like effector nucleases (TALENs) or CRISPR/Cas9. The introduction of a site-specific dsDNA break by the endonuclease activity allows homologous recombination at aberrant target genes, followed by PBase-mediated traceless removal of selectable gene cassettes. This strategy has recently been used in SCD patient derived iPS cells for therapeutic applications [146]. Moreover, PB was also used in conjunction with TALENs to successfully enable seamless correction of mutated β-globin in SCD-specific iPS cells without any detectable off-target activity and undesirable chromosomal alterations [146]. This widens the spectrum of possible therapeutic alternatives for gene therapy against SCD (see above), in particular, based on the approach of gene correction in iPS cells.

The CRISPR/Cas9 system has recently been combined with transposon technology. Typically, CRISPR/Cas9 activity relies on initial sequence complementarity between the target gene in the host genome and the corresponding sgRNA, followed by recognition of the DNA–sgRNA complex by the Cas9 nuclease. For genome editing in iPS cells, humanized Cas9 (*hCas9*) gene under the control of inducible promoter was primarily transposed by PBase activity to ensure stable hCas9-mediated nuclease activity in a time-specific manner upon induction. Then, sgRNA was co-delivered with inducer and contributed to genomic modification in iPS cells. Following transient PBase activity, inducible hCas9 expression cassette was further excised from iPS genome, thereby yielding genome-edited iPS cells with ‘scarless’ transgene removal [144]. Moreover, combinatorial PB-CRISPR/Cas9 platform efficiently enables seamless genetic correction of aberrant genes containing point mutations. This was recently demonstrated in patient-derived iPS cells to correct mutated *HBB* gene for β-thalassemia treatment [147]. In principle, *HBB*-targetting CRISPR/Cas9 components were first transferred into iPS cells to generate DSB break within the gene. Upon CRISPR/Cas9 activity, iPS cells were transfected with a PB transposon carrying selectable marker genes, where both TIRs were flanked by two homologous arms derived from wild-type *HBB* gene. Homologous recombination at cleavage site allowed genetic correction of *HBB* gene, and subsequently PBase activity resulted in traceless excision of PB expression cassette. This results in genetically corrected iPS cells and stable *HBB* gene expression in iPS-derived erythroblasts upon hematopoietic differentiation.

Similarly, PB transposons in conjunction with CRISPR/Cas9 system potentially supports gene therapy in genetic disorders caused by expansion of trinucleotide repeats. As recently shown in iPS cells derived from Huntington’s disease [148], two sgRNAs were designed to remove CAG repeat expansion in exon 1 of *HTT* locus under Cas9 nuclease activity. Homology-directed repair of strand breaks at *HTT* locus was directed by PB transposon harboring integrated selection cassette flanked by two homologous DNA sequences. After successful positive clone isolation, traceless excision of PB selection cassette was achieved by re-introduction of PBase. Collectively, the novel combinatorial PB-CRISPR/Cas9 platform has emerged as a significant advancement in gene editing technology, which allows precise and seamless genetic correction in genome.

## Transposon-based gene therapy in clinical trials

The clinical use of transposon systems for gene transfer was pioneered by Dr Cooper and his team to engineer T cells expressing CAR for T-cell based immunotherapy against CD19-specific B-lineage malignancies including advanced non-Hodgkin lymphoma and acute lymphoblastic leukemia [149]. As an adjuvant therapy, redirected T cells were infused into patients in autologous (NCT00968760)/allogeneic (NCT01497184) settings following HSC transplantation (HSCT) to avert disease relapse due to remaining tumor cells. Currently, the SB11 system has been commonly used for CAR T-cell production by mediating stable expression of CAR in primary T cells following T-cell isolation from peripheral blood. Subsequently, genetically modified T cells were numerically expanded in a selective manner by using artificial APCs (aAPCs) expressing essential co-stimulatory molecules under interleukin (IL)-2 and IL-21 induction [150]. At the end of the enrichment process, CAR-expressing CD3^+^ T cells with ~90% purity could be attained without the presence of any residual SBase activity. This is reassuring from a safety perspective as it would minimize the risk of continued transposon (re)mobilization. Transplantation of autologous CAR T cells into HSCT patients contributed to 83.3 and 100% of progression-free survival (PFS) and overall survival (OS) after 30 months, respectively. Consistently, 53% of PFS and 63% of OS were observed at 12-month post infusion of CAR T cells in allogenic HSCT group. Long-term persistence of circulating CAR T cells was noted, for an average of 201 and 51 days based on autologous and allogeneic redirected T-cell administration, respectively. The decline of PFS, OS, and persistence in allogeneic CAR T-cell infusion supports the use of immunomodulatory molecules to circumvent potential graft-versus-host disease (GVHD), which was absent from the autologous trial. Safety assessment indicated that CAR T cells could be delivered to autologous and allogeneic patients without developing severe GVHD or hypercytokinemia. Collectively, this justifies the clinical use of transposon technology for adoptive T-cell therapy against B-cell malignancies. The same concept of using CD19-targetting CAR T-cell engineering has been implemented into other clinical trials to study its therapeutic efficacy following lymphodepletion (NCT02529813) and for treatment of chronic lymphocytic leukemia (NCT01653717).

Several strategies can be further implemented to improve SB-based CAR T-cell production for adoptive therapy in the clinic. For instance, generation of CAR T cells using SB system for clinical application currently relies on DNA electroporation with high amount of conventional SB transposon/SBase-encoding plasmids, which often leads to cellular toxicity following transfection and yields less numbers of viable cells for further *in vitro* expansion. By employing SB100X technology, it allowed us to use less DNA amount to reach the equivalent transposition efficiency mediated by SB11 upon T-cell transfection [126]. As an alternative, the use of minicircle plasmids to express CARs and SBase significantly increased viability of genetically modified T cells due to the overall reduction in DNA amounts. In addition, minicircle-based engineered T cells required shorter propagation period independent of specific antigen stimulation to obtain sufficient cell number for *in vivo* infusion compared with parental SB transposons [21]. The therapeutic efficacy of CAR T cells could be further optimized to enhance their cytolytic activity and persistence after transplantation. The optimal ratio of CD8^+^ and CD4^+^ CAR-expressing T cells provided synergistic effects which contributed to superior tumor regression *in vivo* compared with when a single cell subpopulation was used [151]. This is consistent with the results obtained from clinical study using CAR T cells with defined subset composition for the treatment of B-cell malignancy (NCT01865617) [152]. In particular, IL-15 signaling through membrane-bound chimeric IL-15 (mbIL15) during the generation of CAR T cells promoted T-cell differentiation into T-memory stem cells, which exhibited more potent antitumor activity and long-term persistence *in vivo* [153].

In addition to the clinical development of T-cell immunotherapy, the SB system has also been explored in the context of a phase I clinical trial of age-related macular degeneration (AMD) gene therapy (project name: ‘TargetAMD’, project ID number: ‘26663-KLV) [154]. AMD is characterized by an aberrant growth of blood vessels into subretinal space due to an overexpression of the vascular endothelial growth factor (VEGF). As a clinical approach for AMD gene therapy, primary retinal pigment epithelial (RPE) and iris pigment epithelial (IPE) cells have been subjected to genetic modification in order to highly express pigment epithelia derived factor (PEDF) – the natural antagonist of VEGF. Transposon-derived miniplasmid devoid of antibiotic resistance marker was chosen as a vehicle for stable integration of the PEDF therapeutic gene in combination with SB100X. This would ultimately offer safety advantages and increase the efficiency of stable gene transfer over the original SB system. Successful genetic engineering of up to 10000 primary RPE and IPE cells derived from multiple donors/patients using this platform underscores the robustness of gene delivery system. The complete assessment of stable PEDF expression and copies number of PEDF in genetically modified target cells supported the safety and efficacy for further development toward clinically graded manufacturing. Currently, the project is under evaluation for approval by Swiss Regulatory Authorities before launching the pilot clinical trial, and the recruitment of patients is aimed to start in September 2017.

## Genome integration profile

Unwanted transgene integration may perturb the expression profiles of oncogenes and tumor suppressor genes and subsequently contribute to oncogenesis. Therefore, integration preferences of transposons have been evaluated to assess their safety profile for use in gene therapy. The preferential target sites of SB and PB transposons are relatively well defined, which are AT palindromes and TTAA tetranucleotides for SB and PB, respectively [155,156]. Particular to SB, the adjacent AT nucleotides flanking the target region favor the bending conformation of DNA, which potentially supports DNA integration [155]. In contrast, potential integration site of *Tol2* transposon is less conserved and composed of TNA(C/G)TTATAA(G/C)TNA sequence [51,157]. Comparative analysis of genomic insertion indicated that the PB transposon displayed the highest tendency of integration toward transcriptional units (~50–55% in RefSeq genes) compared with SB (~25–45% in RefSeq genes) and *Tol2* (~40% in RefSeq genes). In addition, PB and *Tol2* transposons share the insertion bias targetting high GC-content elements, DNase I hypersensitivity regions, as well as transcriptional start sites (TSSs) [83,128,156,157]. Therefore, SB seems to demonstrate a somewhat more favorable integration pattern relative to PB and *Tol2* transposons. These findings are corroborated by a recent genome-wide profiling study showing that the distribution of SB transposon insertions displayed the least deviation from random, hence demonstrated the highest theoretical chance of targetting a safe harbor locus in the human genome compared with PB as well as MLV retrovirus and HIV lentivirus insertions in CD4^+^ T cells with respect to a panel of 40 chromatin states [158]. However, the impact of biased compared with random integration on the risk of insertional oncogenesis should not be overstated as other confounding variables may have a far greater impact, such as the vector design itself. Indeed, despite the presence of DNA integration within transcriptional units, *in vivo* administration of empty transposons in healthy murine models did not contribute to tumor development, supporting the safe use of transposons for gene transfer [43]. Moreover, we also found that robust transposition did not necessarily result in insertional oncogenesis, even in sensitive tumor-prone mouse models suggesting that the vector design itself is critically important [43]. It is prudent to avoid incorporating strong viral enhancers in the transposons to minimize the risk of *cis*-activation of oncogenes that happen to be in proximity of an integrated transposon. Incorporation of insulators into transposon vectors can be considered to prevent the promoter *cis*-activation of neighboring genes and/or epigenetic inactivation of the therapeutic transgene [159–162]. In addition, construction of transposase fusion proteins would allow precise targetted insertion of transposons and improve the integration profiles. By linking a site-specific DNA-binding domain (DBD) to the N- or C- terminus of transposase, transposition could be guided to predetermined regions which are considered ‘safe-harbor’ sites [9]. However, each transposase permits various extents of protein modification. In particular, SB and *Tol2* transposition activity is diminished when transposase ends are linked to DBD, whereas PB retains high integration efficiency upon transposase modification at the protein terminus [18,163,164]. Unlike SB and *Tol2* transposase, redirected PBase allows successful protein modification to achieve up to 50% of on-target insertion [163,164]. The distribution of off-target PB insertion is identified in RefSeq genes (57.8%) and ±5 kb from recognition sites (19.9%) [164]. This indicates the feasibility of DNA-specific targetting by PBase engineering. Head-to-head comparison of off-target activity between CRISPR/Cas9 and redirected PB transposon system on the same target genes is yet to be investigated to determine the most efficient method of gene editing.

## Conclusion and perspectives

DNA transposons enable non-viral stable gene transfer through DNA integration, which could potentially substitute the need of viral vectors. This may be advantageous from the perspective of vector manufacturing and further augment the safety profiles, particularly by minimizing potential immune reactions. However, the oversimplified and widely held assumption that non-viral vectors do not evoke any immune responses at all, does not take into account the possible risks of innate immune system activation due to the DNA itself. Moreover, the therapeutic transgene and the underlying genetic defect in the host greatly affect the immunogenicity of the therapeutic protein. Consequently, immune modulation may still be required to prolong expression of the gene of interest upon stable transposition in the host genome. Typically, two-component transposon platforms have been developed based on SB, PB, and *Tol2* transposons to mediate stable transgene integration into host genome. The use of mRNA encoding transposases is attractive to prevent continued transposon mobilization. Modification of TIRs and generation of hyperactive and codon-optimized transposase variants boosted the overall transposition efficiencies. In particular, the SB100X, hySB100, and hyPBases are amongst the most potent transposases developed to date. Many preclinical studies in disease models that mimic the cognate human disorders underscore the potential of transposons for gene therapy. Nevertheless, some on the non-viral transfection methods would need to be further optimized to boost efficiency while reducing toxicity. This is the case for transfection in CD34^+^ HSC as well as in the case of liver-directed gene transfer. Nevertheless, the use of nanoparticle transfection methods could be considered a promising option for *in vivo* transfer of transposons as it offers high-efficiency genetic modification in target cells (*in casu* hepatocytes and LSECs) without the presence of systemic toxicity. This would need to be validated in large animal models. Recently, the potential of SB for clinical applications was demonstrated in the context of T-cell-based adoptive immunotherapy for lymphoid cancer yielding promising efficacy and safety data. The continued development and refinement of transposon technologies and the first steps toward their clinical translation will likely herald a new and exciting era in gene therapy.

## Abbreviations

AAV: adeno-associated viral
AMD: age-related macular degeneration
APC: antigen presenting cell
CAR: chimeric antigen receptor
CRISPR/Cas9: clustered regularly interspaced short palindromic repeats/CRISPR-associated protein-9
DBD: DNA-binding domain
DMD: Duchenne muscular dystrophy
DSB: double-strand breaks
FVIII: blood clotting factor VIII
FIX: blood clotting factor IX
GAG: glycosaminoglycan
GVHD: graft-versus-host disease
hAT: hobo/Ac/Tam3
hCas9: humanized Cas9
HD: hydrodynamic
Hpx: hemopexin
hPBase: human PB transposase
HSC: hematopoietic stem cell
HSCT: HSC transplantation
hyPBase: hyperactive PBase
hySB100X: hyperactive SB100X
IL: interleukin
IPE: iris pigment epithelial
iPS: induced pluripotent stem
LSEC: liver sinusoidal endothelial cell
MAB: mesoangioblast
mPBase: mouse PB transposase
MPS: mucopolysaccharidosis
MSC: mesenchymal stem cell
OS: overall survival
PB: *piggyBac*
PBase: PB transposase
PEDF: pigment epithelium-derived factor
PEI: polyethyleneimine
PFS: progression-free survival
RPE: retinal pigment epithelial
SB: *Sleeping Beauty*
SBase: SB transposase
SCD: sickle cell disease
sgRNA: single guide RNA
TALEN: transcription activator-like effector nuclease
TIR: terminal inverted repeat
TSD: target-site duplication
VEGF: vascular endothelial growth factor
